# Supplementary material for: The role of phosphorus in the solid electrolyte interphase of argyrodite solid electrolytes
Source: Nat Commun. 2025 Oct 21;16:9304. doi: 10.1038/s41467-025-64357-3 (PMC12540886; doi:10.1038/s41467-025-64357-3)
Supplement: Supplementary file 1 — Supplementary Information [file 41467_2025_64357_MOESM1_ESM.pdf]

# Supplementary Information
## The Role of Phosphorus in the Solid Electrolyte Interphase of Argyrodite Solid Electrolytes

Matthew Burton[1,2], Ben Jagger[1], Yi Liang[1], Joshua S. Gibson[1], Jack Aspinall[1], Zhongdui Long[1,2], Jack E. N. Swallow[1], Robert S. Weatherup[1,3], and Mauro Pasta[1,2*]

[1]Department of Materials, University of Oxford, Parks Road, Oxford OX1 3PH, United Kingdom
[2]The Faraday Institution, Quad One, Harwell Science and Innovation Campus, Didcot OX11 0RA, United Kingdom
[3]Diamond Light Source, Harwell Science and Innovation Campus, Didcot OX11 0DE, United Kingdom
[*]Corresponding author: mauro.pasta@materials.ox.ac.uk

## Supplementary Note 1: Stoichiometric Equations for the Chemical Reduction of $Li_6PS_5Cl$

The complete chemical reduction of $Li_6PS_5Cl$ and all SEI components requires 8 Li atoms, as shown in Equation S1.

$$8Li + Li_6PS_5Cl \rightarrow Li_3P + 5Li_2S + LiCl \tag{S1}$$

Thermodynamically, $Li_6PS_5Cl$ could be chemically reduced with just 5 Li atoms,[1] as shown in Equation S2.

$$5Li + Li_6PS_5Cl \rightarrow P + 5Li_2S + LiCl \tag{S2}$$

Thermodynamically, $Li_6PS_5Cl$ could also be chemically reduced by $Li_3P$, as shown in Equation S3.

$$\frac{5}{3}Li_3P + Li_6PS_5Cl \rightarrow \frac{8}{3}P + 5Li_2S + LiCl \tag{S3}$$

Additionally, $Li_6PS_5Cl$ could also be chemically reduced by $Li_3P$ resulting in a partially reduced P species existing, as shown in Equation S4.

$$Li_3P + \frac{x}{5}Li_6PS_5Cl \rightarrow Li_{3-x}P_{\frac{5+x}{5}} + xLi_2S + \frac{x}{5}LiCl \tag{S4}$$

Thermodynamically, $Li_6PS_5Cl$ could also be chemically reduced by $Li_xP$ (where x <3), as shown in Equation S5.

$$Li_xP + \frac{x}{5}Li_6PS_5Cl \rightarrow \frac{5+x}{5}P + xLi_2S + \frac{x}{5}LiCl \tag{S5}$$

Additionally, $Li_6PS_5Cl$ could also be chemically reduced by $Li_xP$ resulting in a partially reduced P species still existing, as shown in Equation S6, where y<x.

$$Li_xP + \frac{y}{5}Li_6PS_5Cl \rightarrow Li_{x-y}P_{\frac{5+y}{5}} + yLi_2S + \frac{y}{5}LiCl \tag{S6}$$

## Supplementary Note 2: CTTA fitting to the Deal-Grove model

In the Deal-Grove model[2] film thickness, $x_0$, is predicted to increase over time, $t$, according to:

$$x_0^2 + Ax_0 = B(t + \tau) \tag{S7}$$

where A and B are fitting constants and $\tau$ is a time offset allowing for an initial thickness at time $t = 0$.
In the long time limit, when $t >> \frac{A^2}{4B}$, film growth is diffusion limited and Eq. S7 reduces to:

$$x_0 \approx \sqrt{Bt} \tag{S8}$$

Fitting CTTA data from Figure 1b gives a gradient which is equal to $B^{0.5}$. The gradient equals 1.423, which therefore give a $B$ value of 2.025. Fitting the CTTA from Figure 1b to the Deal-Grove model, but fixing $B$ as a value of 2.025 gives a negative $A$ (-12.724) and $\tau$ (-19.988) (Figure S18) which is not physical. This indicates that the fit is not appropriate.
In the short time limit, when $t << \frac{A^2}{4B}$, film growth is limited by reaction kinetics and Eq. S7 reduces to:

$$x_0 \approx \frac{B}{A}(t + \tau) \tag{S9}$$

Fitting the first two CTTA data points as linear gives a gradient and thus $B/A$ of 1.567, thus using the previously deduced $B$ gives an $A$ of 1.292. Taking this $A$ and $B$ would define short time as << 0.21 h. The first plating step in the CTTA alone is 0.1 h, therefore, this would imply the kinetically limited regime is not accessible in the time frame of the CTTA experiment.

## Supplementary Note 3: Transmission Line Model Fitting of the SEI

The SEI is modeled as a long open uniform distributed transmission line, which can be approximated as a $M_a$ element.[3] The relationship between the impedance from a $M_a$ element and the resistance of the SEI ($R_{SEI}$) is shown in Equation S10.

$$Z_{\mathrm{M_a}} = R_{SEI} \frac{\coth\left((\tau j\omega)^{\alpha/2}\right)}{(\tau j\omega)^{\alpha/2}} \tag{S10}$$

# Additional data

**Supplementary Table 1** Volume fractions of the SEI of $Li_6PS_5Cl$ if P is fully lithiated

| Species | Molar Volume ($cm^3$ $mol^{-1}$) | Molar Ratio | Product ($cm^3$ $mol^{-1}$) | Volume Fraction (%) |
|---|---|---|---|---|
| $Li_2S$ | 27.5 | 5 | 137.5 | 70.8 |
| LiCl | 20.5 | 1 | 20.5 | 10.6 |
| $Li_3P$ | 36.2 | 1 | 36.2 | 18.6 |
| Total | | | 194.2 | |

**Supplementary Table 2** Volume fractions of the SEI of $Li_{5.7}PS_{4.7}Cl_{0.65}Br_{0.65}$ if P is fully lithiated

| Species | Molar Volume ($cm^3$ $mol^{-1}$) | Molar Ratio | Product ($cm^3$ $mol^{-1}$) | Volume Fraction (%) |
|---|---|---|---|---|
| $Li_2S$ | 27.5 | 4.7 | 129.3 | 66.3 |
| LiCl | 20.5 | 0.65 | 13.3 | 6.8 |
| LiCl | 20.5 | 0.65 | 16.3 | 8.4 |
| $Li_3P$ | 36.2 | 1 | 36.2 | 18.6 |
| Total | | | 195.1 | |

**Supplementary Table 3** Volume fractions of the SEI of $Li_{5.5}PS_{4.5}Cl_{0.75}Br_{0.75}$ if P is fully lithiated

| Species | Molar Volume ($cm^3$ $mol^{-1}$) | Molar Ratio | Product ($cm^3$ $mol^{-1}$) | Volume Fraction (%) |
|---|---|---|---|---|
| $Li_2S$ | 27.5 | 4.5 | 123.8 | 63.7 |
| LiCl | 20.5 | 0.75 | 15.4 | 7.9 |
| LiCl | 20.5 | 0.75 | 18.8 | 9.7 |
| $Li_3P$ | 36.2 | 1 | 36.2 | 18.6 |
| Total | | | 194.2 | |

**Supplementary Table 4** Volume fractions of the SEI of $Li_6PS_5Cl$ if P is not lithiated

| Species | Molar Volume ($cm^3$ $mol^{-1}$) | Molar Ratio | Product ($cm^3$ $mol^{-1}$) | Volume Fraction (%) |
|---|---|---|---|---|
| $Li_2S$ | 27.5 | 5 | 137.5 | 80.4 |
| LiCl | 20.5 | 1 | 20.5 | 12.0 |
| P | 13 | 1 | 13 | 7.6 |
| Total | | | 171.0 | |

**Supplementary Table 5** Fitting parameters and fit quality data not otherwise plotted of the growing SEI PEIS

| Accumulated Charge (μA h $cm^{-2}$) | t (s) | Q (nF $s^{\alpha-1}$) | $\alpha$ | $\alpha$**2** | $\chi^2$ $\|\mathbf{Z}\|^{-1}$ |
|---|---|---|---|---|---|
| 1.56 | 7.488E-5 | 24.06 | 0.9003 | 0.6323 | 0.00486 |
| 3.12 | 1.946E-4 | 18.5 | 0.9241 | 0.6535 | 0.00475 |
| 4.68 | 2.336E-4 | 34.23 | 0.8721 | 0.6585 | 0.00196 |
| 6.24 | 4.19E-4 | 25.98 | 0.8943 | 0.6684 | 0.00433 |
| 7.8 | 5.665E-4 | 50.27 | 0.8332 | 0.6771 | 0.00824 |
| 9.35 | 7.003E-4 | 16.88 | 0.9378 | 0.6741 | 0.00268 |
| 10.9 | 9.8E-4 | 14.22 | 0.9536 | 0.6843 | 0.00805 |
| 12.5 | 0.00119 | 22.8 | 0.9118 | 0.6913 | 0.01348 |
| 14 | 0.00138 | 15.64 | 0.9503 | 0.6922 | 0.01118 |
| 15.6 | 0.0013 | 19.64 | 0.9348 | 0.6832 | 0.00487 |
| 17.1 | 0.00159 | 19.37 | 0.9384 | 0.69 | 0.00868 |
| 18.7 | 0.00199 | 18.23 | 0.9445 | 0.6989 | 0.01536 |
| 20.3 | 0.00216 | 19.09 | 0.9387 | 0.6988 | 0.01563 |
| 21.8 | 0.00252 | 18.72 | 0.943 | 0.7037 | 0.02281 |
| 23.4 | 0.0023 | 22.7 | 0.9291 | 0.6933 | 0.01358 |
| 24.9 | 0.00251 | 24.41 | 0.928 | 0.6937 | 0.0168 |
| 26.5 | 0.00272 | 16.11 | 0.9636 | 0.693 | 0.00903 |
| 28.1 | 0.00269 | 27.36 | 0.9205 | 0.6897 | 0.0159 |
| 29.6 | 0.00262 | 20.99 | 0.9502 | 0.6811 | 0.01031 |
| 31.2 | 0.00286 | 33.48 | 0.9142 | 0.6853 | 0.01764 |
| 32.7 | 0.00281 | 23.62 | 0.9438 | 0.6775 | 0.01066 |
| 34.3 | 0.00312 | 26.36 | 0.9421 | 0.6811 | 0.01668 |
| 35.9 | 0.0034 | 43.29 | 0.901 | 0.6836 | 0.02181 |
| 37.4 | 0.00349 | 47.66 | 0.8952 | 0.6808 | 0.0238 |
| 39 | 0.00364 | 50.1 | 0.8945 | 0.6789 | 0.00679 |
| 40.5 | 0.00379 | 30.99 | 0.9361 | 0.6738 | 0.02111 |
| 42.1 | 0.00405 | 62.51 | 0.8789 | 0.6789 | 0.03087 |
| 43.7 | 0.00377 | 46.45 | 0.9074 | 0.6682 | 0.04869 |
| 45.2 | 0.00414 | 47.61 | 0.9067 | 0.6706 | 0.02442 |
| 46.8 | 0.00377 | 86.67 | 0.8564 | 0.6635 | 0.02239 |
| 48.3 | 0.00432 | 52.86 | 0.9003 | 0.6679 | 0.024 |
| 49.9 | 0.00402 | 61.59 | 0.8888 | 0.6604 | 0.01922 |

**Supplementary Table 6** Fitting parameters and fit quality data not otherwise plotted of the OCV SEI PEIS

| OCV time (h) | t (s) | Q (nF $s^{\alpha-1}$) | $\alpha$ | $\alpha$**2** | $\chi^2$ $\|\mathbf{Z}\|^{-1}$ |
|---|---|---|---|---|---|
| 0 | 0.00402 | 61.59 | 0.8888 | 0.6604 | 0.01922 |
| 25 | 0.00403 | 93.56 | 0.8574 | 0.6689 | 0.02222 |
| 50 | 0.00479 | 75.96 | 0.8751 | 0.6437 | 0.0061 |
| 75 | 0.00326 | 68.54 | 0.8831 | 0.6369 | 0.0052 |
| 100 | 0.00297 | 71.2 | 0.8789 | 0.6219 | 0.00898 |
| 125 | 0.004 | 63.82 | 0.8885 | 0.6539 | 0.00697 |
| 150 | 0.00368 | 67.29 | 0.885 | 0.6459 | 0.006 |
| 175 | 0.00375 | 71.57 | 0.8802 | 0.6497 | 0.00618 |
| 200 | 0.00348 | 74.68 | 0.8781 | 0.6469 | 0.0062 |
| 225 | 0.0036 | 71.4 | 0.8816 | 0.6512 | 0.00638 |
| 250 | 0.00318 | 66 | 0.8878 | 0.6392 | 0.0057 |
| 275 | 0.00328 | 64.64 | 0.8903 | 0.6483 | 0.00668 |
| 300 | 0.00297 | 47.76 | 0.9147 | 0.6437 | 0.01538 |
| 325 | 0.00306 | 51.86 | 0.9044 | 0.6481 | 0.01597 |
| 350 | 0.00312 | 37.1 | 0.9295 | 0.6468 | 0.01163 |
| 375 | 0.00295 | 40.76 | 0.9223 | 0.6469 | 0.01273 |
| 400 | 0.00288 | 52.44 | 0.9048 | 0.6611 | 0.02028 |

**Supplementary Table 7** XPS fitting parameters after VEP on a $Li_6PS_5Cl$ pellet, corresponding to Fig. 3a and Supplementary Fig. S6.

| Transition | Species | Binding Energy (eV) | FWHM (eV) | Spin-Orbit Splitting (eV) |
|---|---|---|---|---|
| S 2p | $Li_6PS_5Cl$ | $161.4 \pm 0.1$ | $1.15 \pm 0.08$ | 1.2 |
| | $Li_2S$ | $160.1 \pm 0.1$ | $1.13 \pm 0.03$ | 1.2 |
| P 2p | $Li_6PS_5Cl$ | $132.0 \pm 0.1$ | $1.90 \pm 0.01$ | 0.9 |
| | $Li_xP$ | $128.9 \pm 0.1$ | $1.7 \pm 0.2$ | 0.9 |
| | $Li_yP$ | $127.2 \pm 0.1$ | $1.7 \pm 0.2$ | 0.9 |
| | $Li_3P$ | 125.8 | 1.51 | 0.9 |

**Supplementary Table 8** XPS fitting parameters during VEP on a phosphorus-coated $Li_6PS_5Cl$ pellet, corresponding to Fig. 4a.

| Transition | Species | Binding Energy (eV) | FWHM (eV) | Spin-Orbit Splitting (eV) |
|---|---|---|---|---|
| S 2p | P−S−P | 162.6 | 1.15 | 1.2 |
| | $Li_6PS_5Cl$ | $161.4 \pm 0.1$ | $1.10 \pm 0.02$ | 1.2 |
| | $Li_2S$ | $160.1 \pm 0.1$ | $1.10 \pm 0.02$ | 1.2 |
| P 2p | P−O | $133.1 \pm 0.1$ | $1.4 \pm 0.2$ | 0.9 |
| | $Li_6PS_5Cl$ | $131.8 \pm 0.1$ | $1.4 \pm 0.2$ | 0.9 |
| | $P^0$ | $129.1 \pm 0.2$ | $1.4 \pm 0.2$ | 0.9 |
| | $Li_xP$ | $127.7 \pm 0.1$ | $1.4 \pm 0.2$ | 0.9 |
| | $Li_yP$ | $126.9 \pm 0.1$ | $1.4 \pm 0.2$ | 0.9 |
| | $Li_3P$ | $125.7 \pm 0.1$ | $1.5 \pm 0.2$ | 0.9 |

**Supplementary Table 9** SOXPES and HAXPES fitting parameters from a pristine $Li_6PS_5Cl$ pellet, corresponding to Supplementary Fig. S10.

| Transition | Species | Binding Energy (eV) | FWHM (eV) | Spin-Orbit Splitting (eV) |
|---|---|---|---|---|
| | | **SOXPES** | | |
| O *1s* | $Li_2CO_3$ | 531.6 | 1.43 | – |
| | LiOH | 530.7 | 1.43 | – |
| Cl *2p* | $Li_6PS_5Cl$ | 198.5 | 1.10 | 1.6 |
| P *2s* | $Li_6PS_5Cl$ | 189.4 | 1.18 | – |
| S *2p* | $Li_2SO_4$ | 166.7 | 0.81 | 1.2 |
| | $Li_6PS_5Cl$ | 161.3 | 0.81 | 1.2 |
| | $Li_2S$ | 160.0 | 0.81 | 1.2 |
| P *2p* | $Li_6PS_5Cl$ | 131.9 | 0.68 | 0.9 |
| Li *1s* | $Li_6PS_5Cl$ | 55.7 | 1.36 | – |
| | | **2.2 keV** | | |
| O *1s* | $Li_2CO_3$ | 531.6 | 1.43 | – |
| | LiOH | 530.6 | 1.43 | – |
| Cl *2p* | $Li_6PS_5Cl$ | 198.5 | 1.11 | 1.6 |
| P *2s* | $Li_6PS_5Cl$ | 189.3 | 1.35 | – |
| S 2p | $Li_2SO_4$ | 166.7 | 0.82 | 1.2 |
| | $Li_6PS_5Cl$ | 161.2 | 0.82 | 1.2 |
| | $Li_2S$ | 159.9 | 0.82 | 1.2 |
| P *2p* | $Li_6PS_5Cl$ | 131.5 | 0.70 | 0.9 |
| Li *1s* | $Li_6PS_5Cl$ | 55.2 | 1.42 | – |
| | | **6.6 keV** | | |
| O *1s* | $Li_2CO_3$ | 531.7 | 1.38 | – |
| | LiOH | 530.7 | 1.38 | – |
| Cl *2p* | $Li_6PS_5Cl$ | 198.5 | 1.03 | 1.6 |
| P *2s* | $Li_6PS_5Cl$ | 189.4 | 1.41 | – |
| S 2p | $Li_2SO_4$ | 166.6 | 0.81 | 1.2 |
| | $Li_6PS_5Cl$ | 161.2 | 0.81 | 1.2 |
| | $Li_2S$ | 159.9 | 0.81 | 1.2 |
| P *2p* | $Li_6PS_5Cl$ | 131.5 | 0.68 | 0.9 |
| Li *1s* | $Li_6PS_5Cl$ | 55.4 | 1.38 | – |

**Supplementary Table 10** SOXPES and HAXPES fitting parameters after approximately 20 nm of Li was evaporated onto a $Li_6PS_5Cl$ pellet (0 h / 6 h / 12 h), corresponding to Fig. 5a, 5c and Supplementary Fig. S11.

| Transition | Species | Binding Energy (eV) | FWHM (eV) | Spin-Orbit Splitting (eV) |
|---|---|---|---|---|
| | | **SOXPES** | | |
| O *1s* | $Li_2CO_3$ | 531.8 / 531.6 / 531.6 | 1.40 / 1.63 / 1.59 | – |
| | LiOH | 530.7 / 530.6 / 530.7 | 1.40 / 1.63 / 1.59 | – |
| | Oxide | 529.1 / – / – | 1.42 / – / – | – |
| | $Li_2O$ | 528.1 / 528.1 / 528.2 | 1.42 / 1.46 / 1.39 | – |
| Cl *2p* | $Li_6PS_5Cl$/LiCl | 198.5 / 198.5 / 198.5 | 1.14 / 1.12 / 1.12 | 1.6 |
| S 2p | $Li_2SO_4$ | 166.5 / – / – | 0.92 / – / – | 1.2 |
| | $Li_6PS_5Cl$ | 161.3 / 161.4 / 161.3 | 0.92 / 0.91 / 0.90 | 1.2 |
| | $Li_2S$ | 160.0 / 160.1 / 160.0 | 0.92 / 0.91 / 0.90 | 1.2 |
| Li *1s* | $Li^+$ | 53.4 / 53.8 / 53.9 | 1.75 / 1.83 / 1.78 | – |
| | $Li^0$ | 52.2 / 52.2 / 52.2 | 0.35 / 0.35 / 0.34 | – |
| | | **2.2 keV** | | |
| O *1s* | $Li_2CO_3$ | 531.7 / 531.6 / 531.8 | 1.88 / 1.64 / 1.61 | – |
| | LiOH | 530.6 / 530.7 / 530.7 | 1.88 / 1.64 / 1.61 | – |
| | $Li_2O$ | 528.2 / 528.2 / 528.2 | 1.34 / 1.24 / 1.21 | – |
| Cl *2p* | $Li_6PS_5Cl$/LiCl | 198.5 / 198.5 / 198.5 | 1.14 / 1.10 / 1.09 | 1.6 |
| S 2p | $Li_6PS_5Cl$ | 161.2 / 161.2 / 161.2 | 0.87 / 0.88 / 0.88 | 1.2 |
| | $Li_2S$ | 160.0 / 160.0 / 160.0 | 0.87 / 0.88 / 0.88 | 1.2 |
| P 2p | $Li_6PS_5Cl$ | 131.6 / 131.6 / 131.5 | 0.73 / 0.78 / 0.90 | 0.9 |
| | $Li_xP$ | 129.7 / 129.6 / 129.1 | 0.73 / 0.78 / 0.90 | 0.9 |
| | $Li_yP$ | 128.0 / 127.8 / 127.2 | 0.73 / 0.78 / 0.90 | 0.9 |
| | $Li_3P$ | 126.1 / 125.7 / 125.5 | 0.73 / 0.78 / 0.90 | 0.9 |
| Li *1s* | $Li^+$ | 53.7 / 53.8 / 53.8 | 1.40 / 1.54 / 1.61 | – |
| | $Li^0$ | 52.1 / 52.1 / 52.1 | 0.46 / 0.47 / 0.45 | – |
| | | **6.6 keV** | | |
| O *1s* | $Li_2CO_3$ | 531.8 / 531.6 / 531.6 | 1.48 / 1.51 / 1.45 | – |
| | LiOH | 530.7 / 530.6 / 530.6 | 1.48 / 1.51 / 1.45 | – |
| | $Li_2O$ | 528.2 / 528.1 / 528.1 | 1.31 / 1.23 / 1.22 | – |
| Cl *2p* | $Li_6PS_5Cl$/LiCl | 198.5 / 198.5 / 198.5 | 1.04 / 1.07 / 1.14 | 1.6 |
| P 2s | $Li_6PS_5Cl$ | 189.4 / 189.4 / 189.4 | 1.53 / 1.55 / 1.72 | – |
| | $Li_xP$ | 187.3 / 187.2 / 186.9 | 1.53 / 1.55 / 1.72 | – |
| | $Li_yP$ | 186.0 / 186.0 / 185.6 | 1.53 / 1.55 / 1.72 | – |
| | $Li_3P$ | 184.2 / 183.9 / 183.7 | 1.53 / 1.55 / 1.72 | – |
| S 2p | $Li_6PS_5Cl$ | 161.2 / 161.2 / 161.2 | 0.85 / 0.86 / 0.86 | 1.2 |
| | $Li_2S$ | 159.9 / 159.9 / 159.9 | 0.85 / 0.86 / 0.86 | 1.2 |
| Li *1s* | $Li^+$ | 54.0 / 54.1 / 54.1 | 1.64 / 1.58 / 1.51 | – |
| | $Li^0$ | 52.2 / 52.2 / 52.2 | 0.52 / 0.56 / 0.57 | – |

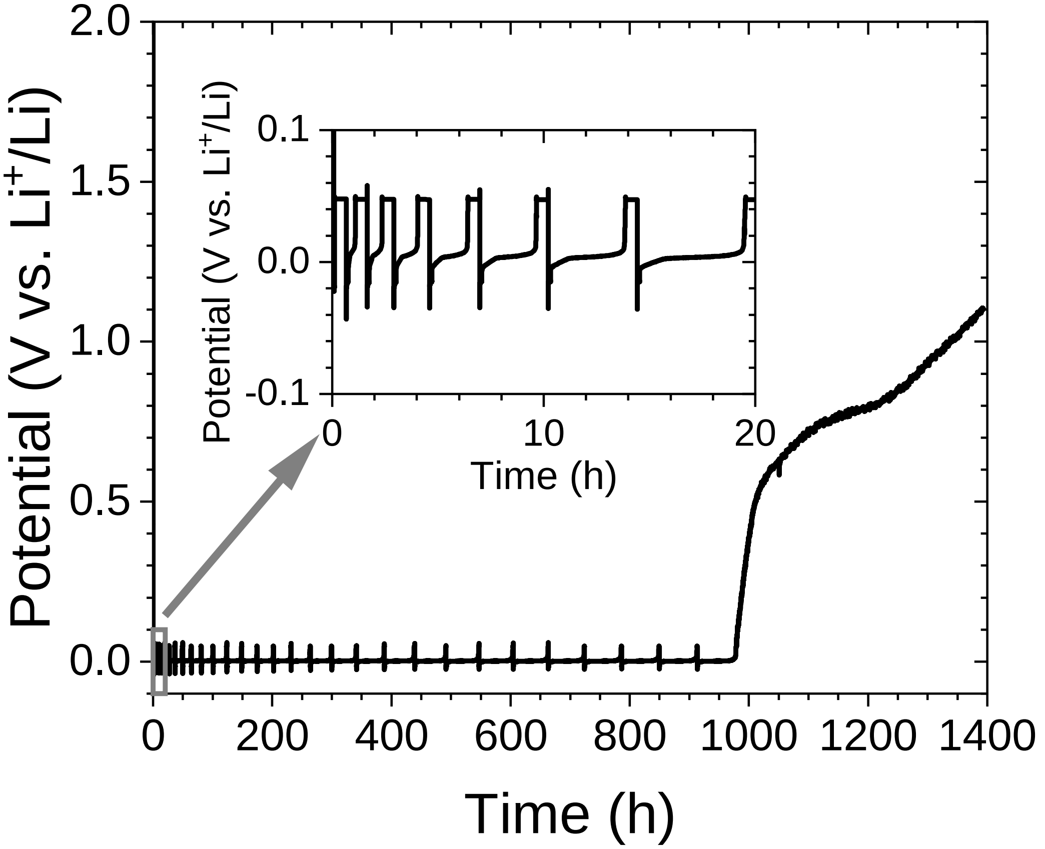


Figure S1: Three electrode CTTA measurement with PEIS conducted once a 50 mV OCV was reached (inset shows the initial cycles).

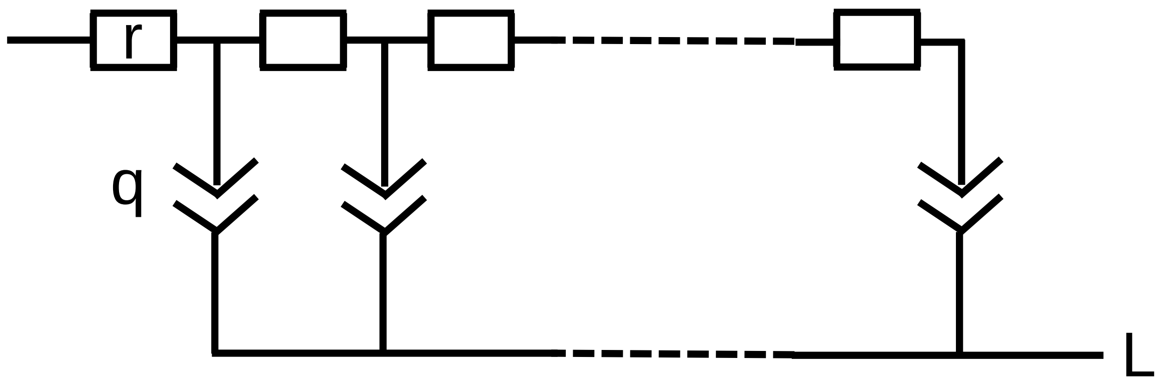


Figure S2: Equivalent circuit of the transmission line element; open-circuited uniform distributed resistor constant phase element (URQ), which is approximated as a $M_a$.[3] The resistance (R) of the transmission line is equal to the length (L) of the transmission line times by the resistivity (r) of the transmission line. This R is taken as the $R_{SEI}$ in this work

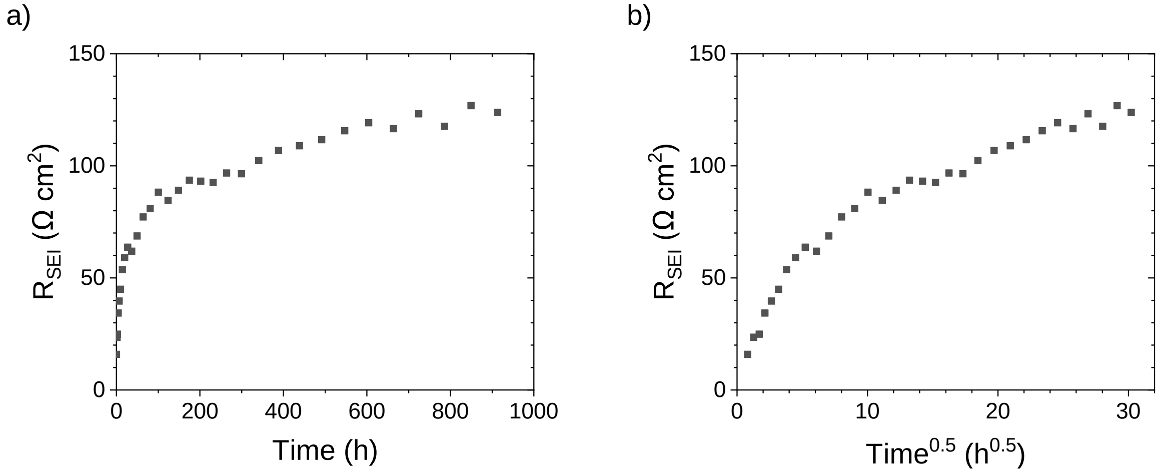


Figure S3: The increase in the SEI resistance against CTTA time, plotted with a) linear time and b) the square root of time. Source data are provided as a Source Data file.

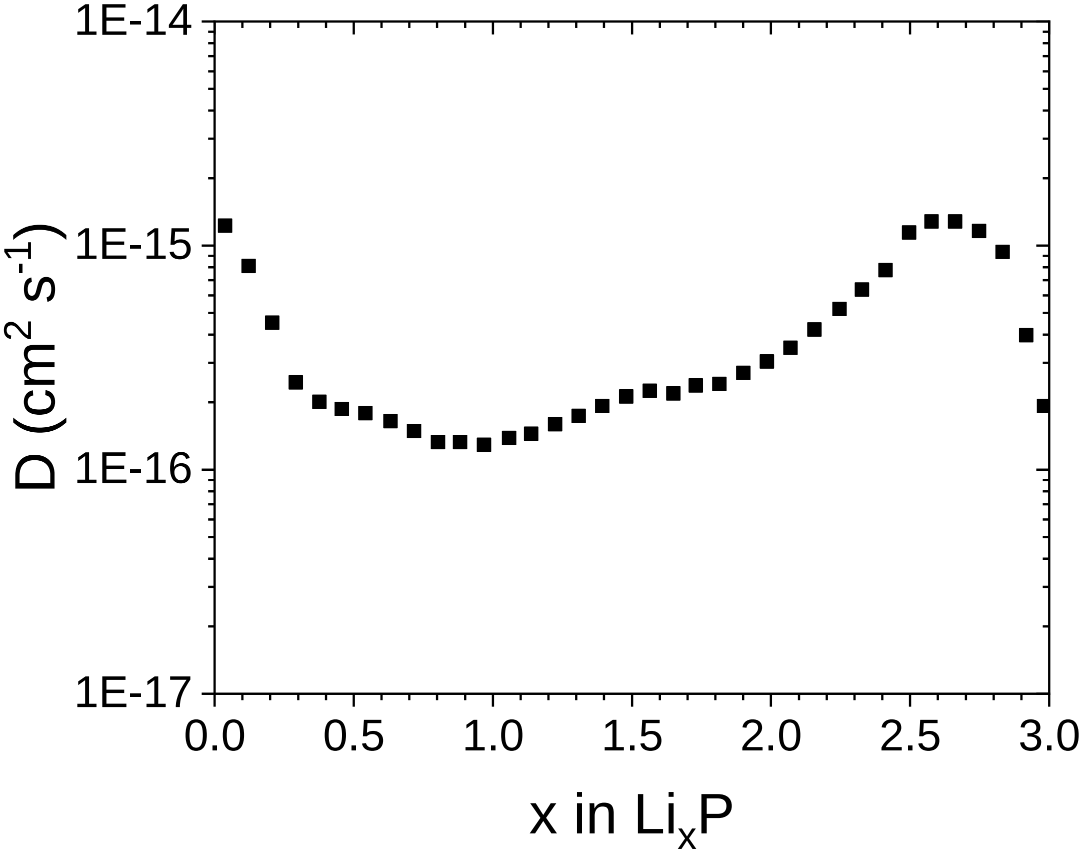


Figure S4: The suggested diffusivity of Li in $Li_xP$ from GITT studies as reported by Capone *et al.*[4] Source data are provided as a Source Data file.

.

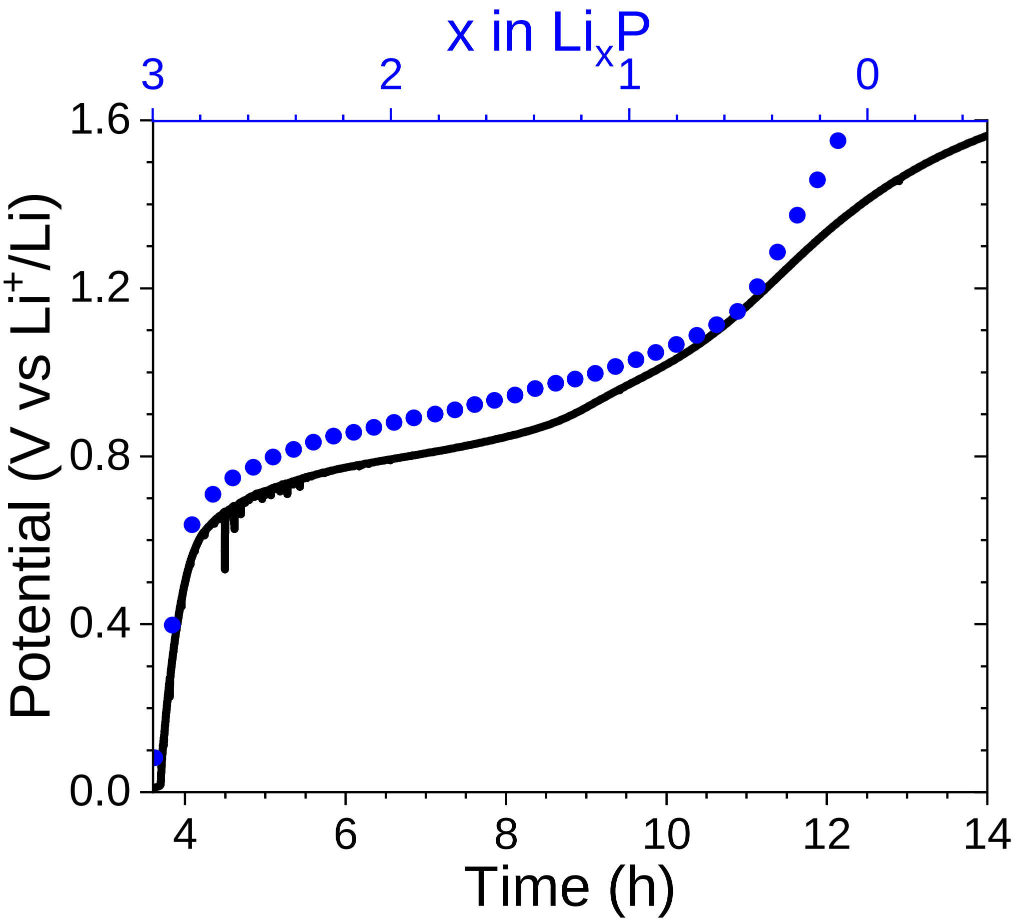


Figure S5: OCV times between 3.6 h and 14 h after 0.01 mAh of lithium was electroplated through a 1 cm diameter cylindrical pellet of $Li_6PS_5Cl$ over 1 hour (black line), compared to delithiation profile of red phosphorous from Capone *et al.* (blue circles).[4]

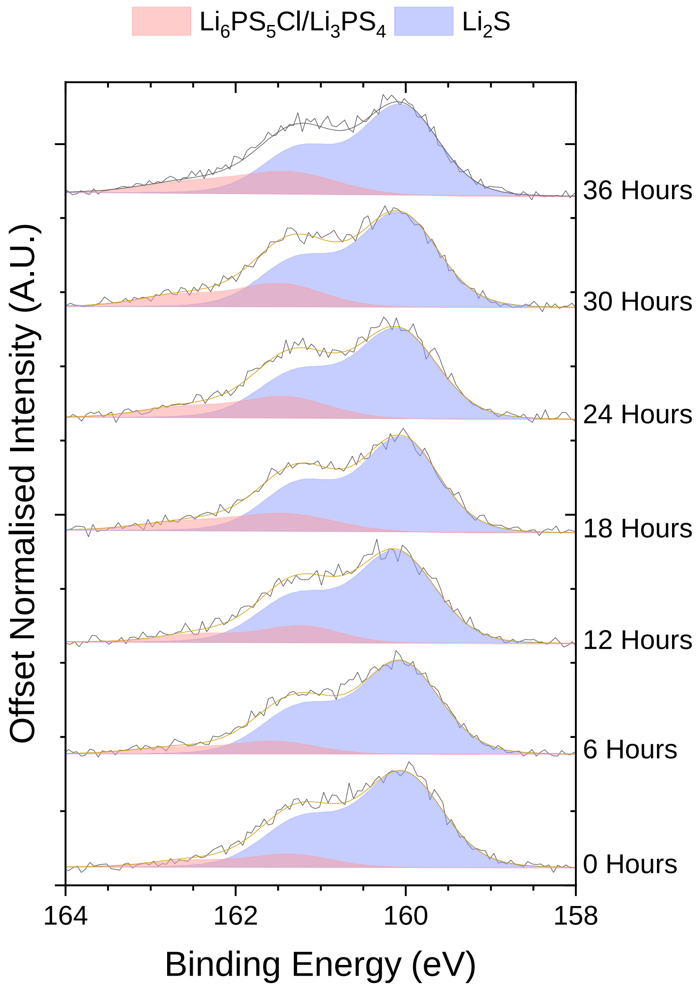


Figure S6: The sulphur *2p* XPS data every 6 hours with the fitted components after ∼0.01 mAh cm$^{-2}$ of lithium was VEP through a $Li_6PS_5Cl$ pellet over 1 hour using an ebeam current of 2.5 µA (∼0.01 mA cm$^{-2}$). Source data are provided as a Source Data file.

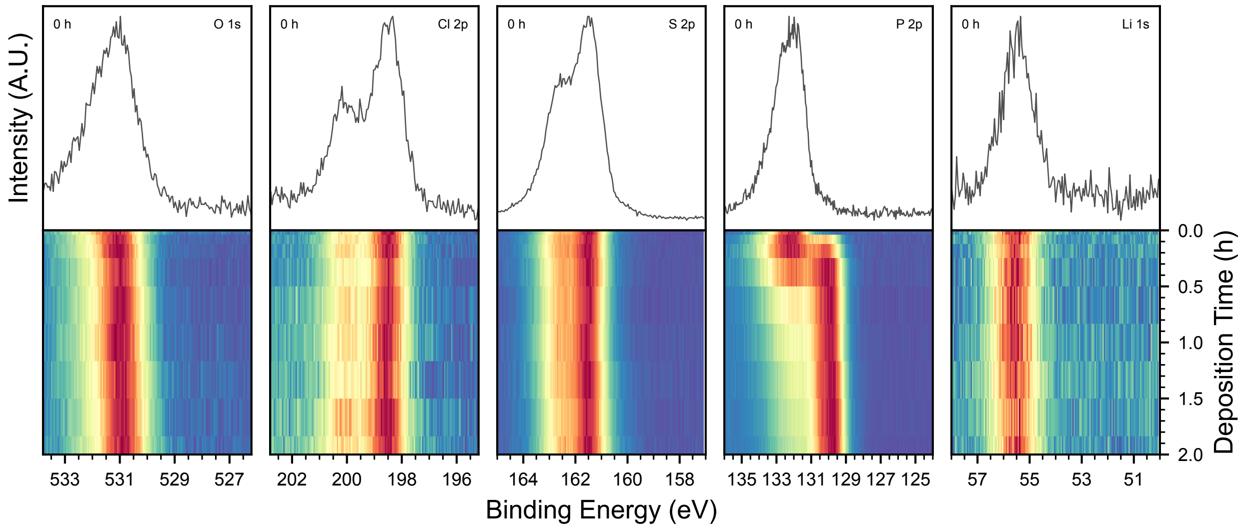


Figure S7: Normalised O *1s*, Cl 2p, S 2p, P *2p* and Li *1s* XPS spectra gathered during phosphorus sputtering onto a $Li_6PS_5Cl$ pellet for 2 h. Source data are provided as a Source Data file.

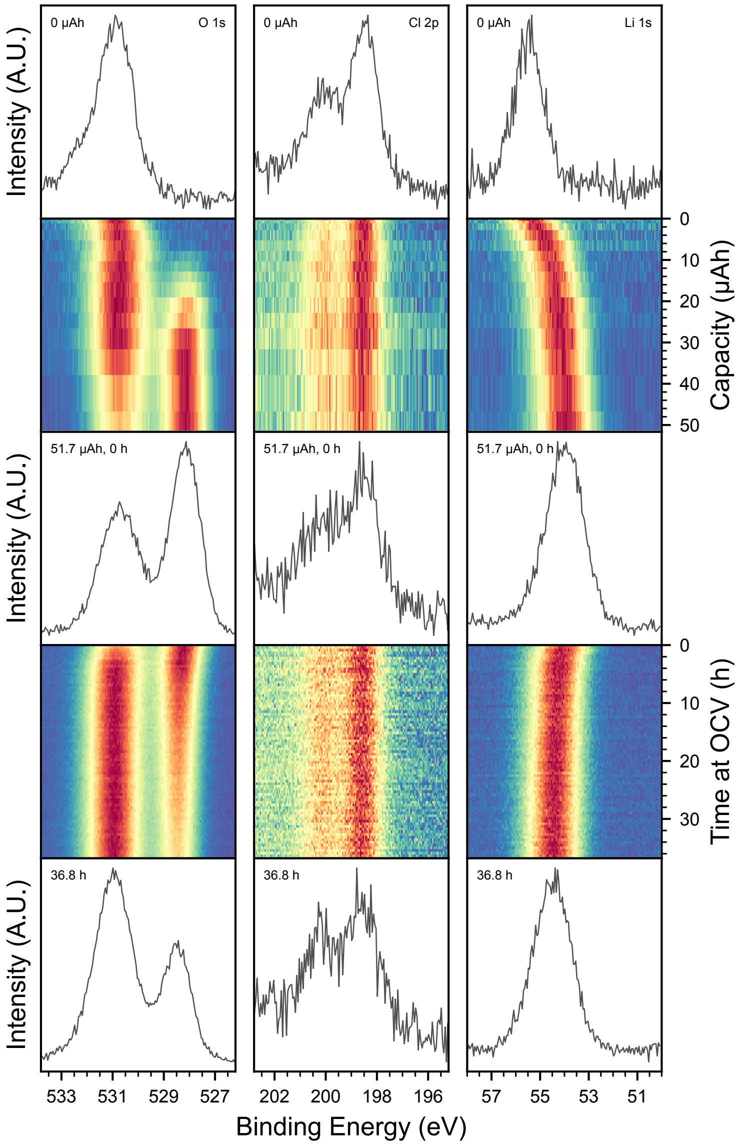


Figure S8: Evolution of the O 1s, Cl *2p* and Li *1s* spectra during VEP-XPS lithiation of a sputtered phosphorus layer on top of a $Li_6PS_5Cl$ pellet using an ebeam current of 30 µA to a capacity of 51.7 µAh (∼0.26 mAh cm$^{-2}$), followed by resting under OCV for 36.8 h. All spectra have been normalised for clarity. Source data are provided as a Source Data file.

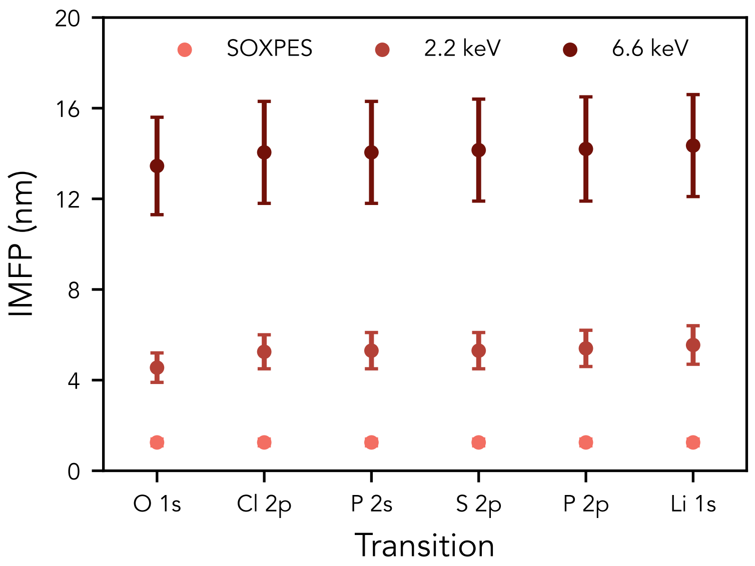


Figure S9: Electron inelastic-mean-free-paths (IMFPs) for each transition and incident photon energy calculated using the TPP-2M formula [5]. Upper and lower bounds are estimated by calculating the IMFP in $Li^0$ and $Li_6PS_5Cl$, respectively, whilst the center point represents the mean between these. Source data are provided as a Source Data file.

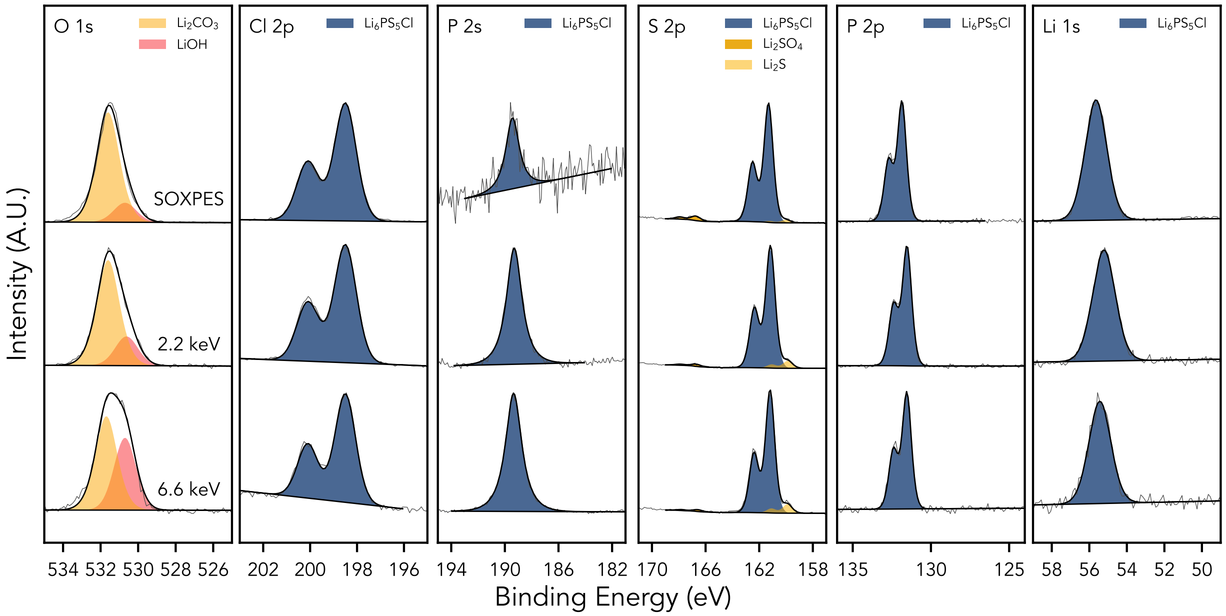


Figure S10: Full O 1s, Cl 2p, P 2s, S 2p, P *2p* and Li *1s* SOXPES and HAXPES spectra from a pristine $Li_6PS_5Cl$ pellet. Photoelectrons emitted during SOXPES have a kinetic energy of 315 eV, and HAXPES was performed with incident photon energies of 2.2 and 6.6 keV. Source data are provided as a Source Data file.

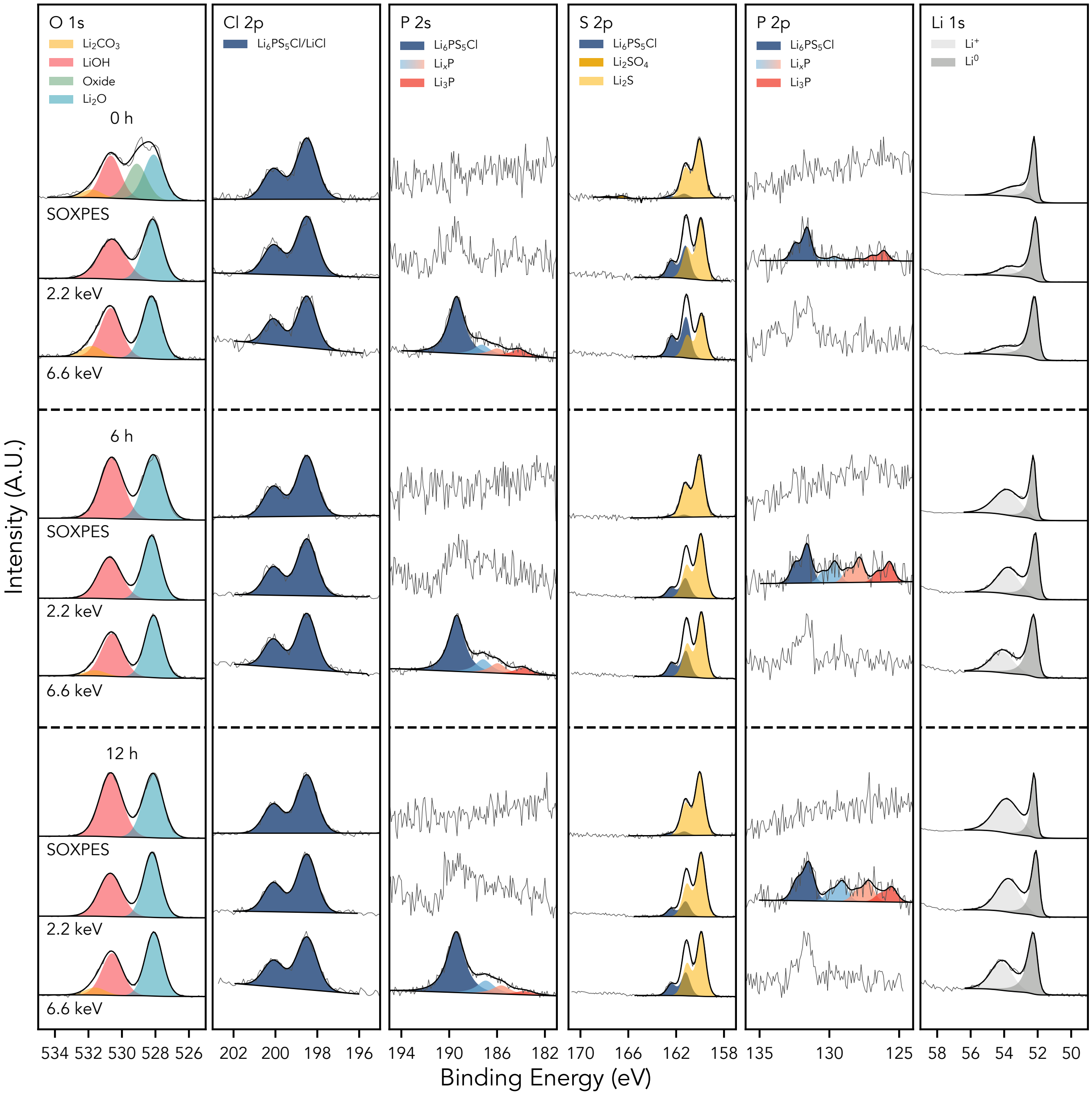


Figure S11: Full O 1s, Cl 2p, P 2s, S 2p, P *2p* and Li *1s* SOXPES and HAXPES spectra after approximately 20 nm of Li was evaporated onto a $Li_6PS_5Cl$ pellet. Photoelectrons emitted during SOXPES have a kinetic energy of 315 eV, and HAXPES was performed with incident photon energies of 2.2 and 6.6 keV. Measurements were performed 0, 6 and 12 h after Li evaporation. Source data are provided as a Source Data file.

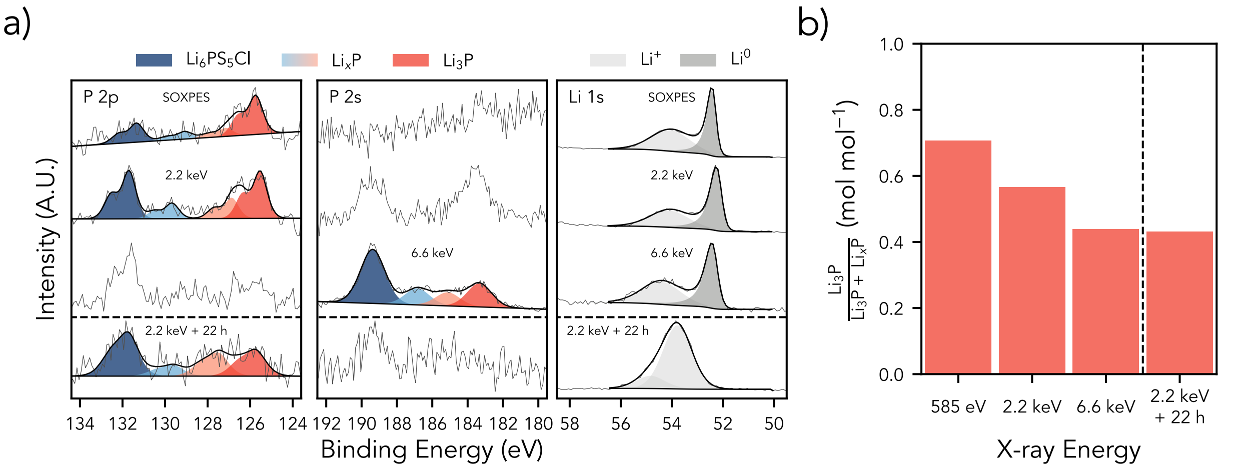


Figure S12: Soft and hard X-ray photoelectron spectroscopy (SOXPES and HAXPES) on an $Li_6PS_5Cl$ pellet with ~20 nm of Li evaporated on top. a) P 2p, P *2s* and Li *1s* spectra gathered using SOXPES (photon energies of 585, 645 and 505 eV for the P 2p, P *2s* and Li *1s* regions, respectively) and HAXPES with photon energies of 2.2 and 6.6 keV. HAXPES with 2.2 keV photons was additionally performed following a 22 h waiting period where all $Li^0$ had reacted, as evident from the disappearance of the $Li_0$ peak. The P *2p* spectra display the best signal-to-noise ratio for the SOXPES and 2.2 keV HAXPES data, while the P *2s* spectrum is best for the 6.6 keV HAXPES data as the photoionization cross section of the P *2s* orbital exceeds that of the P *2p* orbital at high incident photon energies [6]. These spectra were therefore used for subsequent analysis, as denoted by the fitted peaks. b) mole fraction of $Li_3P$ in the P-containing SEI species calculated from the spectra in a), demonstrating a reduction in proportion of $Li_3P$ with increasing probing depth and increasing time. Source data are provided as a Source Data file.

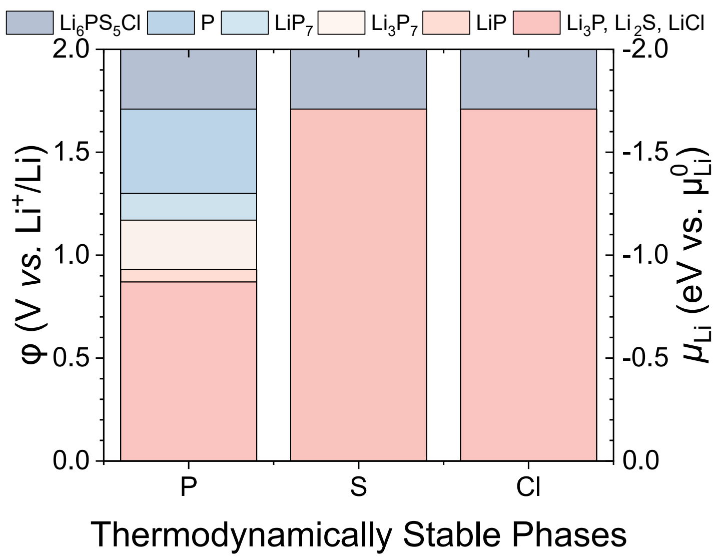


Figure S13: Thermodynamic stabilities of the elemental components of $Li_6PS_5Cl$ in the reductive region,[1].

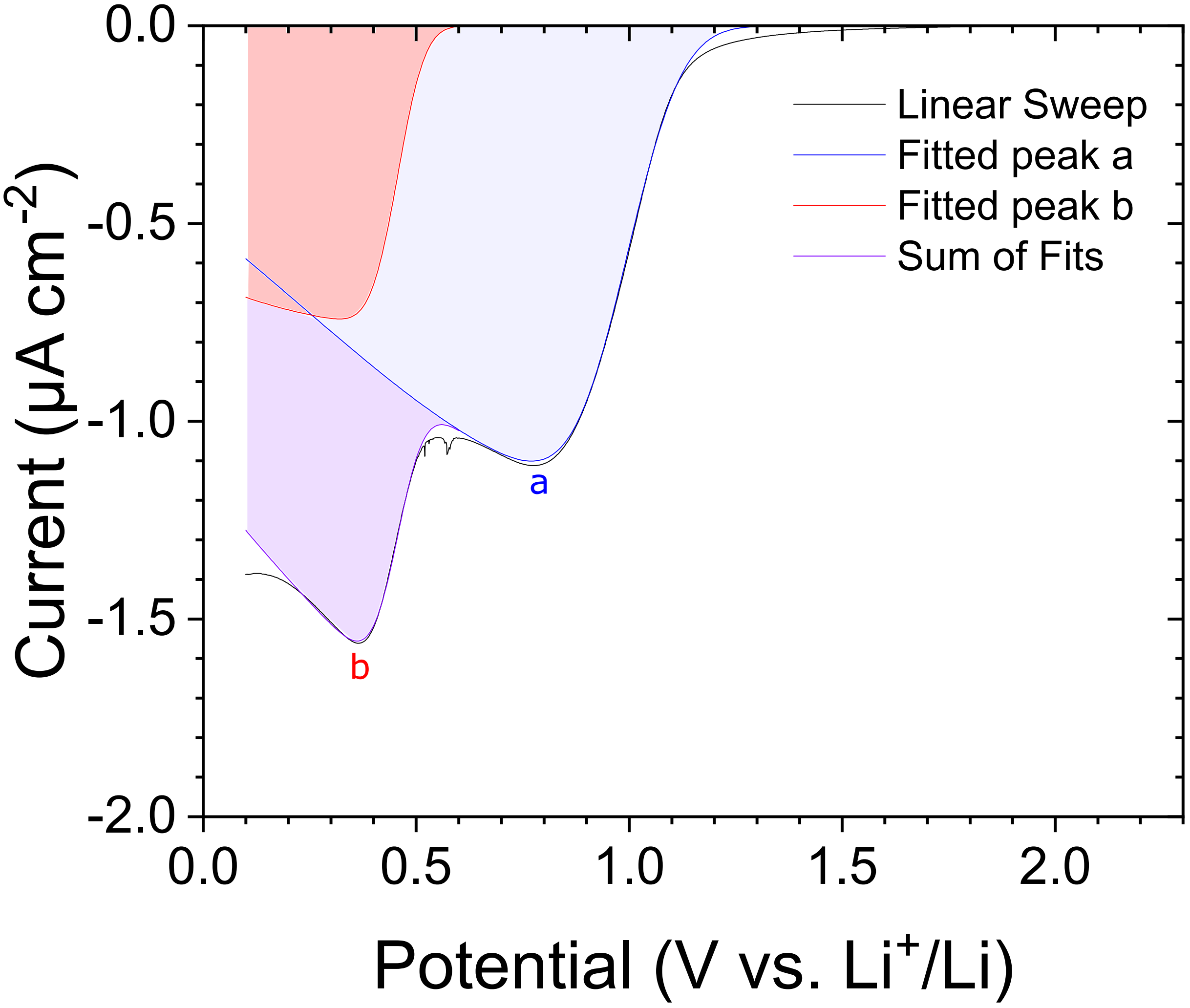


Figure S14: The initial linear sweep of the cyclic voltammetry, with peaks fitted to skewed Gaussians. The area under peak a corresponds to 2.36 $\mu$Ah cm$^{-2}$, whilst the area under peak b corresponds to 0.72 $\mu$Ah cm$^{-2}$. The total capacity of 3.08 $\mu$Ah cm$^{-2}$ would correspond to an SEI thickness of 28 nm, if it was fully dense and consisted of $Li_2S$, LiCl and $Li_3P$. Source data are provided as a Source Data file.

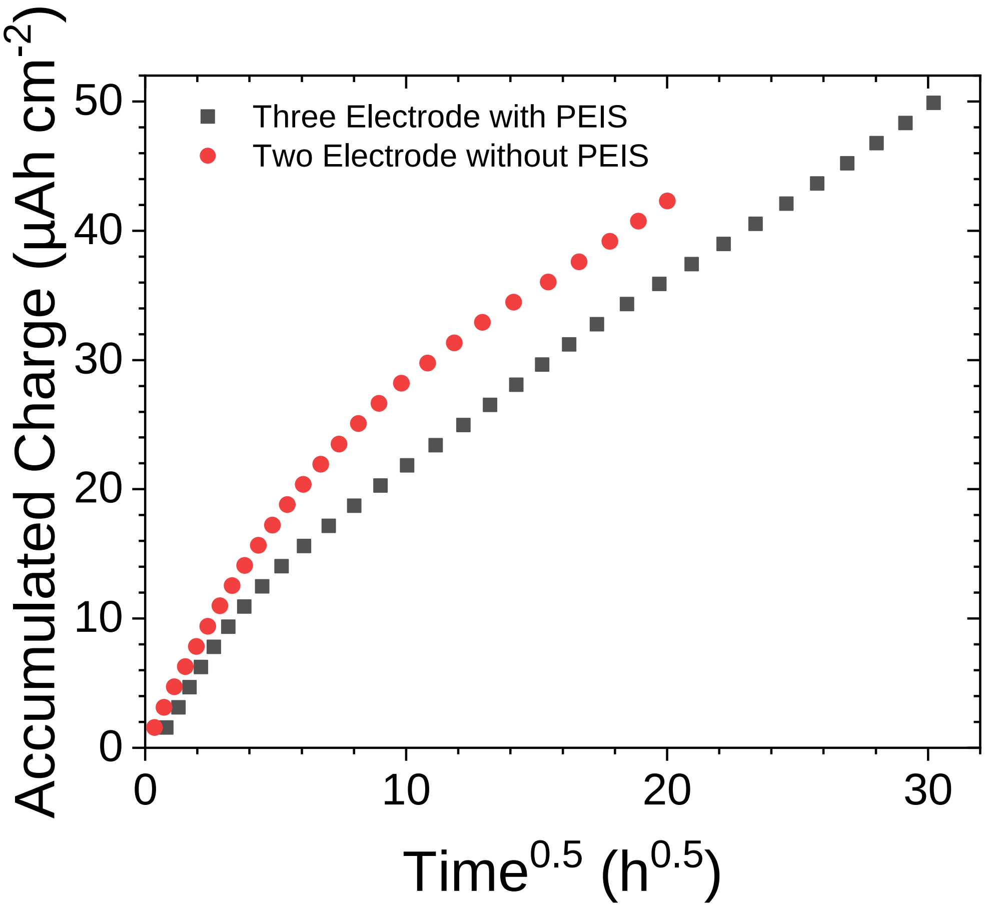


Figure S15: A comparison between three electrode and two electrode CTTA measurements. The three electrode cell conducted PEIS once a 50 mV OCV was reached before applying more reducing current, whilst the two electrode cell did not conduct PEIS and applied more reducing current as soon as 50 mV OCV was reached. Each plating step was -1.56 $\mu$A cm$^{-2}$ for 10 minutes. The cell pressure was 13 MPa and the temperature 303 K. Source data are provided as a Source Data file.

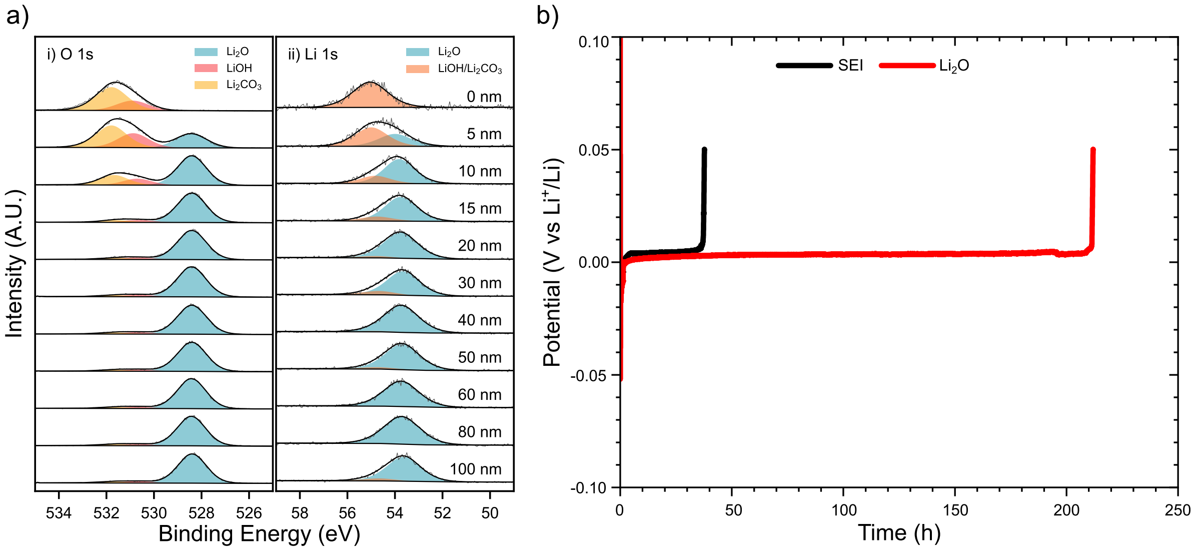


Figure S16: Rate of SEI formation through a preexisting SEI and a native passivation layer. a) Depth profiling of a 100 nm layer of thermally evaporated Li left in an Ar glovebox for two weeks (i, oxygen spectra; ii, Li spectra) revealing that except for a surface carbonate the entire film has turned into an oxide, b) the time taken for 0.02 mAh $cm^{-2}$ (∼100 nm) of electroplated Li to react through SEI formed from 0.02 mAh $cm^{-2}$ (∼100nm) of Li reacting with $Li_6PS_5Cl$ (black) and $Li_2O$ formed from allowing a 100 nm layer of thermally evaporated Li to react naturally in a Ar glovebox (<0.01 ppm of $O_2$ and $H_2O$) for 2 weeks (red). Source data are provided as a Source Data file.

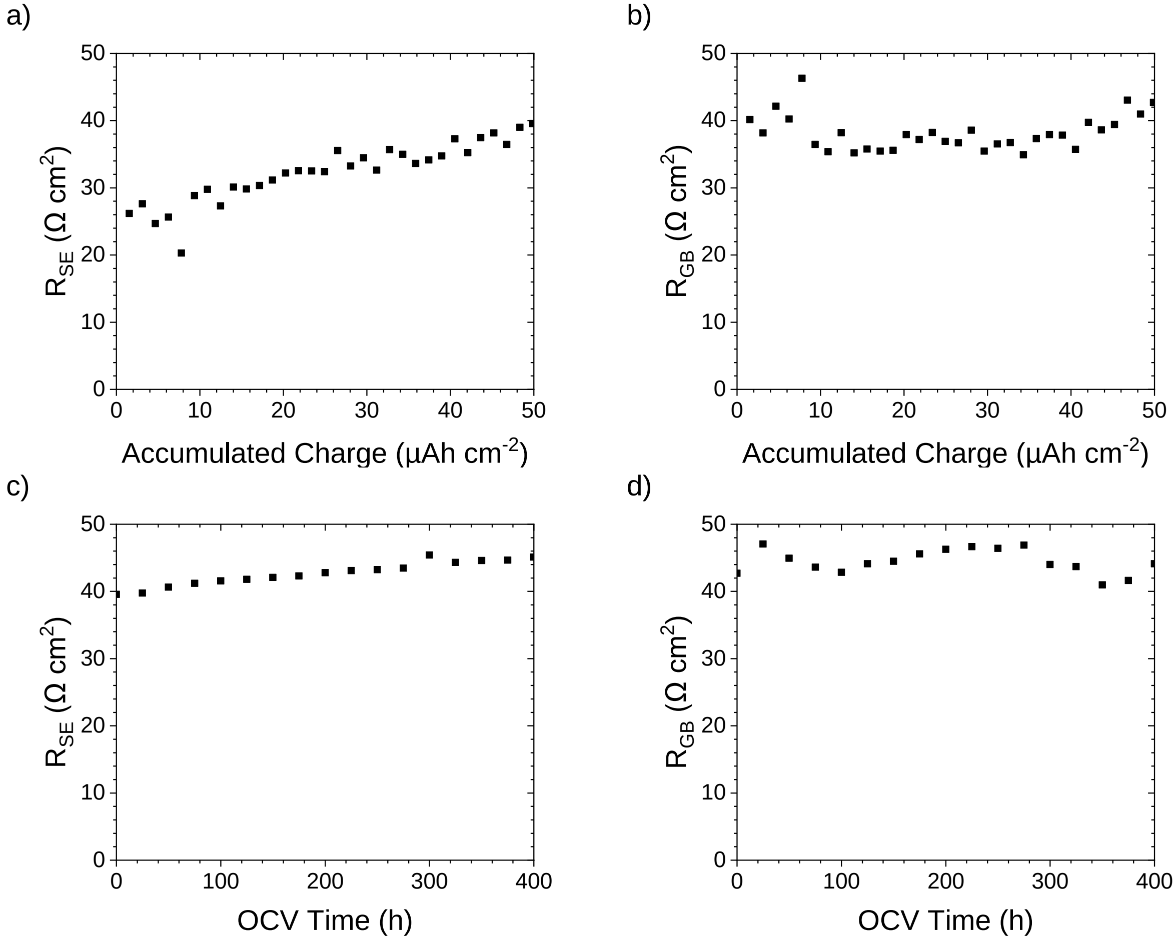


Figure S17: Bulk solid electrolyte (a,c) and grain boundary (b,d) resistance values obtained from fits of the impedance data shown in Figure 2c (a,b) and Figure 3a (c,d). The bulk solid electrolyte resistance ($R_{SE}$) is seen to slightly increase over the course of the measurement, which could be due to a slight degradation of the solid electrolyte over time through reaction with the reference electrode. Source data are provided as a Source Data file.

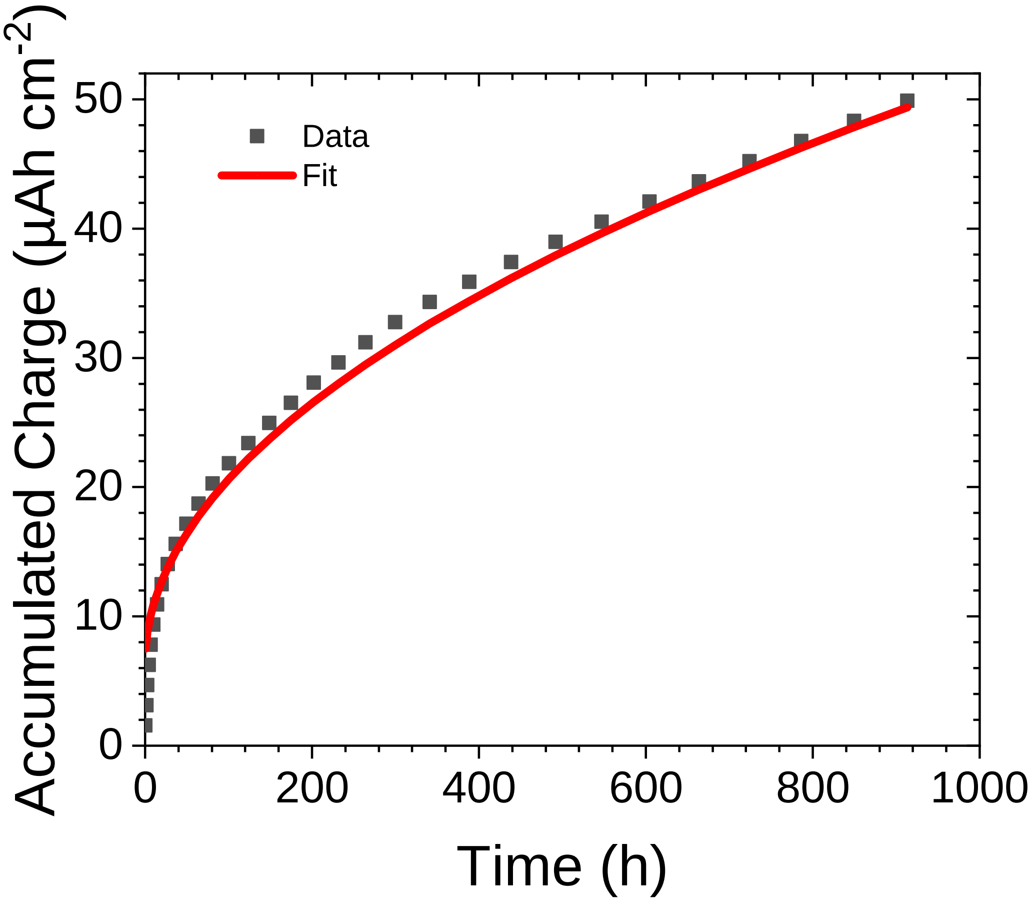


Figure S18: Fitting the CTTA data from Figure 1b to the Deal-Grove model,[2] with a fixed B of 2.025 (deduced from the fit line in Figure 1b) and allowing an A and $\tau$ to be deduced that optimise the fit. A was found to be -12.724, whilst $\tau$ was -19.988.

# References

1. Zhu, Y., He, X. & Mo, Y. Origin of Outstanding Stability in the Lithium Solid Electrolyte Materials: Insights from Thermodynamic Analyses Based on First-Principles Calculations. *ACS Applied Materials and Interfaces* **7,** 23685–23693 (2015).

2. Deal, B. E. & Grove, A. S. General Relationship for the Thermal Oxidation of Silicon. *Journal of Applied Physics* **36,** 3770–3778 (1965).

3. BioLogic. *How to fit transmission lines with ZFit (EIS transmission lines) Battery – Application Note 43*

4. Capone, I. *et al.* Electrochemo-Mechanical Properties of Red Phosphorus Anodes in Lithium, Sodium, and Potassium Ion Batteries. *Matter* **3,** 2012–2028 (2020).

5. Tanuma, S., Powell, C. J. & Penn, D. R. Calculations of electron inelastic mean free paths. IX. Data for 41 elemental solids over the 50 eV to 30 keV range. *Surf. Interface Anal.* **43,** 689–713 (2011).

6. Yeh, J. J. & Lindau, I. Atomic subshell photoionization cross sections and asymmetry parameters: $1 \leq Z \leq 103$. *At. Data Nucl. Data Tables* **32,** 1–155 (1985).
